# Multimodal investigation of electronic transport in PTMA and its impact on organic radical battery performance

**DOI:** 10.1038/s41598-023-37308-5

**Published:** 2023-07-06

**Authors:** Davis Thomas Daniel, Steffen Oevermann, Souvik Mitra, Katharina Rudolf, Andreas Heuer, Rüdiger-A. Eichel, Martin Winter, Diddo Diddens, Gunther Brunklaus, Josef Granwehr

**Affiliations:** 1 Institute of Energy and Climate Research (IEK-9), Forschungszentrum Jülich GmbH, 52425 Jülich, Germany; 2 Institute of Technical and Macromolecular Chemistry, RWTH Aachen University, 52056 Aachen, Germany; 3 Helmholtz Institute Münster (IEK-12), Forschungszentrum Jülich GmbH, 48149 Münster, Germany; 4 Institute of Physical Chemistry, University of Münster, 48149 Münster, Germany; 5 MEET Battery Research Center, University of Münster, 48149 Münster, Germany; 6 Institute of Physical Chemistry, RWTH Aachen University, 52056 Aachen, Germany

**Keywords:** Electrochemistry, Physical chemistry, Polymer chemistry, Theoretical chemistry

## Abstract

Organic radical batteries (ORBs) represent a viable pathway to a more sustainable energy storage technology compared to conventional Li-ion batteries. For further materials and cell development towards competitive energy and power densities, a deeper understanding of electron transport and conductivity in organic radical polymer cathodes is required. Such electron transport is characterised by electron hopping processes, which depend on the presence of closely spaced hopping sites. Using a combination of electrochemical, electron paramagnetic resonance (EPR) spectroscopic, and theoretical molecular dynamics as well as density functional theory modelling techniques, we explored how compositional characteristics of cross-linked poly(2,2,6,6-tetramethyl-1-piperidinyloxy-4-yl methacrylate) (PTMA) polymers govern electron hopping and rationalise their impact on ORB performance. Electrochemistry and EPR spectroscopy not only show a correlation between capacity and the total number of radicals in an ORB using a PTMA cathode, but also indicates that the state-of-health degrades about twice as fast if the amount of radical is reduced by 15%. The presence of up to 3% free monomer radicals did not improve fast charging capabilities. Pulsed EPR indicated that these radicals readily dissolve into the electrolyte but a direct effect on battery degradation could not be shown. However, a qualitative impact cannot be excluded either. The work further illustrates that nitroxide units have a high affinity to the carbon black conductive additive, indicating the possibility of its participation in electron hopping. At the same time, the polymers attempt to adopt a compact conformation to increase radical–radical contact. Hence, a kinetic competition exists, which might gradually be altered towards a thermodynamically more stable configuration by repeated cycling, yet further investigations are required for its characterisation.

## Introduction

Organic radical polymers represent a synergistic combination of polymers and pendant radical moieties, and find extensive use in organic radical polymer batteries (ORBs)^1,2^. The substitution of metals with organic polymers leads to an environmentally sustainable energy storage technology that simultaneously offers high mechanical flexibility, stability, and safer disposal. ORBs exhibit excellent rate performance on account of fast electron transfer between the redox units and a high material activity, rendering them a viable alternative to conventional batteries with metal ion based cathodes^3^. Most ORB research focuses on cells with a lithium metal anode, termed Li-ORBs, which enable benchmarking with existing battery technology, although all-organic batteries, utilising organic polymers as both cathode and anode, have also been reported^4–6^. Amongst organic polymers, radical polymers with pendant redox units outperform conjugated polymers. While conjugated polymers exhibit a sloping voltage during charge/discharge, organic radical polymers provide a stable redox potential, with charge localised on the pendant redox units. The organic radical polymer poly(2,2,6,6-tetramethyl-1-piperidinyloxy-4-yl methacrylate) (PTMA)^7,8^ has become a standard active material in ORBs, owing to favourable electrochemical properties and stability of its monomer, 2,2,6,6-Tetramethylpiperidin-1-oxyl (TEMPO) methacrylate^9^. PTMA is typically employed as cathode material in Li-ORBs, providing a discharge cell voltage of 3.5 V and a theoretical discharge capacity of $C_\text {theo}= 111~{\hbox {mAh}}\,{\hbox {g}}^{-1}$ for a one-electron redox reaction^10,11^.

**Figure 1:**
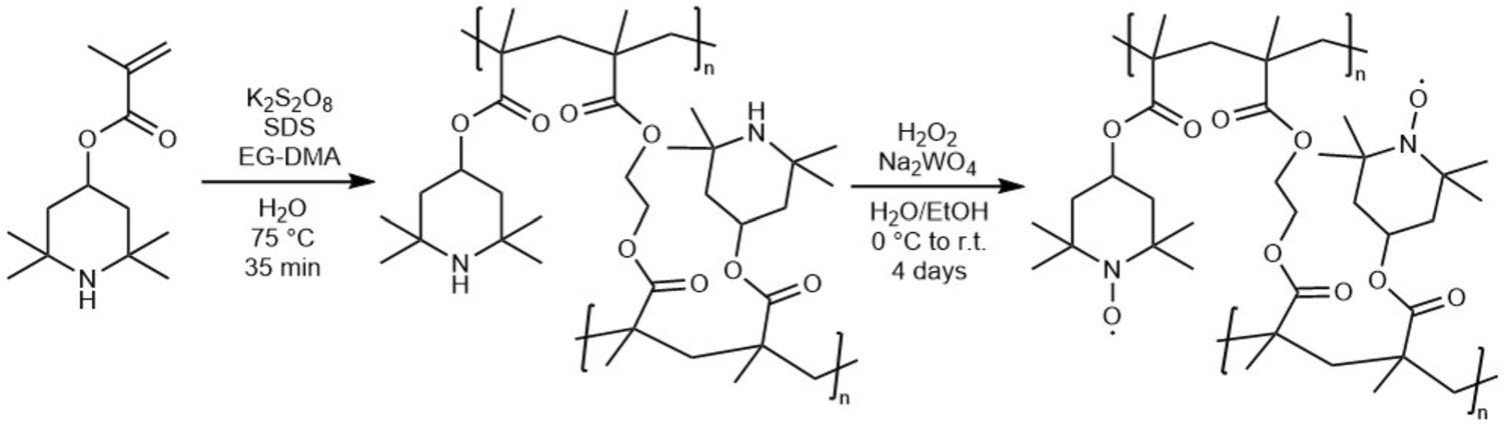
Synthetic route for cross-linked PTMA, consisting of an emulsion polymerisation and an oxidation reaction with ${\hbox {H}_2\hbox {O}_2}$, as previously published by Münch *et al.*^43^.

Electron transport in PTMA and other organic radical polymers with non-conjugated backbones is facilitated by electron hopping^3,12–14^. The diffusion coefficient of such a process is $D = k_\text {a}k_\text {hop}\delta ^{2}C/6$, where $k_\text {a}$ is the association constant (0.23 $\text {M}^{-1}$ for TEMPO^15^), $k_\text {hop}$ is the electron hopping rate, $\delta$ is the distance between redox units, and *C* is the total redox unit concentration^12,16,17^. To study $k_\text {hop}$ between two redox centres, Marcus theory can be invoked ^18,19^. Rates $k_\text {hop}$ are distance dependent and often decay exponentially with increasing separation $\delta$^20^. Therefore, a high radical packing density is desired for continuous hopping pathways. Theoretical models of PTMA oligomer films without solvent reported a distance between nitrogen atoms required for hopping of 0.4–0.7 nm^21^. Radical separation on the polymer chain and, therefore, electron hopping, can be affected by several factors. An incomplete oxidation of redox units during synthesis (see Fig. 1) can cause an uneven distribution of radicals along the backbone^22^, increasing the separation of radicals and decreasing the probability of electron hopping. Polymer swelling in electrolytes is another factor which may lead to an increased radical separation, decreasing inter-chain electron hopping and conductivity^12^.

At the cost of a lower energy density, long-range conductivity can be improved by adding conductive additives, such as carbon black (CB), which interconnect different regions of active material. As most organic radical polymers such as PTMA feature insulating backbones and possess low intrinsic conductivity^23^, using conductive additives is common practice, and significant amounts up to 70 wt% have been reported in ORBs^24^. Another strategy is to utilise flexible polymer backbones, which increase the short-range conductivity through the formation of local percolation networks of nitroxide radicals^25^. Because of incomplete polymerisation, contributions from free monomer radicals and low molecular weight oligomers were observed even in PTMA polymers with a high degree of polymerisation^26^. These free monomer radicals, which are not tethered to the polymer backbone, can also provide additional sites for electron hopping. The conductivity of PTMA films was reported to increase by almost a factor two upon addition of up to 5 wt% of monomer units as dopants^27^. Another study reported a similar conductivity enhancement by adding a TEMPO based redox-active salt as dopant^28^. An improved rate performance is therefore expected due to the dopants, but a systematic electrochemical study with varying amounts of free monomer radicals is required to ascertain such a performance improvement in ORBs. Conversely, as for low molecular weight and linear polymers, additional dopants may undergo dissolution in the electrolyte and may not participate in electron hopping with the primary conductive network^29^. To suppress dissolution of low molecular weight polymers, cross-linking is commonly used.^29^ However, cross-linking may not be effective in preventing dissolution in case of radicals which are not tethered. Consequently, the connectivity in an electron hopping pathway which involves free monomer radicals as additional hopping sites, is affected. Conductivity improvements afforded by such dopants may not be realised unless their dissolution is suppressed. However, an evaluation of capacity fading attributed to such free monomer radicals has not been attempted yet.

Previous Molecular Dynamics (MD) studies of PTMA were performed either for the solid state^21^ or in the presence of solvents such as acetonitrile^19^. The main focus of these studies was to understand the structural properties of PTMA in such systems and estimate the electron transfer rates, whereas PTMA in a complex environment such as an electrolyte has not been simulated so far. A PTMA-in-electrolyte simulation would provide insights into radical separation in the presence of electrolyte and aid in understanding the ionic and electronic transport in such a system.

Electron paramagnetic resonance (EPR) spectroscopy, a technique which detects unpaired electron spins, is well suited for investigating battery materials^30–33^. In ORB research, continuous wave (CW) EPR is routinely used for radical quantification^8,34^, while pulsed EPR can be used to probe specific interactions between the ORB components^35^. Furthermore, *in operando* and *in situ* EPR techniques have also been applied to ORB systems^36–38^. Nitroxide radicals are particularly amenable to EPR techniques, evident from their extensive use as spin labels for studying structure and dynamics in biological systems^39,40^. PTMA consists of closely spaced nitroxide radicals undergoing spin exchange^41^, therefore the solution EPR spectrum consists of a single line with a spin-exchange dependent line width. In contrast, dilute nitroxide solutions show a CW EPR spectrum consisting of three lines, arising from hyperfine interaction of the electron spin and the $^{14}$N nuclear spin. As the spectral signature of nitroxide radicals is dependent on their packing density, EPR techniques can, with high sensitivity, distinguish between radicals undergoing exchange and isolated radicals. Pulsed EPR techniques are applicable when the system is magnetically dilute, as the spin relaxation must be long enough to detect a spin echo, which is fulfilled by isolated radicals. Analysis of EPR parameters is often aided by electronic structure methods such as Hartree–Fock, coupled cluster and density functional theory (DFT), which find extensive use in the validation of experimentally obtained magnetic parameters. DFT methods are widely exploited in the field of EPR for calculation of magnetic parameters and establishing structure–parameter correlations^42^.

In this work, we utilise cross-linked PTMA polymer samples, synthesised using established protocols, to quantify the amounts of isolated radicals while investigating the extent to which such free monomer radicals impact the electrochemical performance of ORBs. CW EPR spectroscopy is applied to differentiate between the contributions of radicals undergoing exchange and isolated radicals, while pulsed EPR is performed to probe the interaction of the isolated radicals with CB and the electrolyte. Galvanostatic cycling and rate capability tests were executed to investigate the correlation between the amount of isolated radicals and electrochemical performance. A DFT validated MD simulation methodology is applied to study PTMA dynamics in the presence of electrolyte, and to gain a better understanding of the transport of ions and electrons inside PTMA electrodes.

## Methods

### Materials

4-Methacryloyloxy-2,2,6,6-tetramethylpiperidin-1-oxyl (TEMPO methacrylate , *Sigma–Aldrich*, 97 %), acetonitrile (*Sigma–Aldrich*, anhydrous, 99.8%), *N*-methyl-2-pyrrolidone (NMP, *Sigma–Aldrich*), toluene (anhydrous, 99.8% , *Sigma–Aldrich*), 2,2,6,6-Tetramethyl-4-piperidyl methacrylate (TMPMA, *TCI*), sodium dodecyl sulfate (SDS, *Sigma–Aldrich*), ethylene glycol dimethacrylate (EG-DMA, *TCI*), potassium persulfate (${\hbox {K}_2\hbox {S}_2\hbox {O}_8}$, *Sigma–Aldrich*, $\ge$ 99 %), hydrogen peroxide (${\hbox {H}_2\hbox {O}_2}$, *Sigma–Aldrich*, 30 % in ${\hbox {H}_2\hbox {O}}$), disodium ethylenediamine tetraacetate dihydrate (EDTA, *Sigma–Aldrich*, $\ge$ 99 %), sodium tungstate dihydrate (${\hbox {Na}_2\hbox {WO}_4\cdot2\hbox {H}_2\hbox {O}}$, *Sigma–Aldrich*, $\ge$ 99 %), carboxymethyl cellulose (CMC, *Dow Wolff Cellulosics*), $\hbox {SuperC}65^{\textcircled {C}}$ carbon black (CB, *Imerys Graphite*, *Alfa Aesar*), lithium hexafluorophosphate (${\hbox {LiPF}_6}$, *E-Lyte*), ethylene carbonate (EC, *E-Lyte*) and ethyl methyl carbonate (EMC, *E-Lyte*) , 1 M ${\hbox {LiPF}_6}$ in EC/DMC (50/50 by volume, *Sigma–Aldrich*) were used as received.

### Synthesis

Four cross-linked PTMA samples (labelled I–IV) were prepared by following a two-step synthesis route, including an emulsion polymerisation and an oxidation reaction, as previously reported by Münch *et al.* (see synthetic route in Fig. 1)^43^. The sole deviation from the reported synthesis procedure occurred in PTMA sample III. Here, the oxidation step was terminated after the second ${\hbox {H}_2\hbox {O}_2}$ addition to generate a sample with a less dense radical packing. The details of the synthesis procedure described below refer to sample IV and are exemplary for the other syntheses, with the exception of the lower ${\hbox {H}_2\hbox {O}_2}$ addition for sample III. In addition to samples I–IV, a linear PTMA polymer was fabricated by performing the aforementioned synthesis route without a cross-linker.

#### Emulsion polymerisation

In 250 mL water, TMPMA (15.750 g, 69.90 mmol, 1.000 eq.) and SDS (0.740 g, 2.55 mmol, 0.036 eq.) were dissolved and flushed with nitrogen gas for 30 min while stirring. After heating to $75~^\circ$C for 45 min, EG-DMA (0.417 g, 2.10 mmol, 0.030 eq.) and a deoxygenated solution of ${\hbox {K}_2\hbox {S}_2\hbox {O}_8}$ (0.262 g, 0.97 mmol, 0.014 eq.) in 10 ml water were added to the emulsion. Subsequently, the solution was heated to $75~^\circ$C for 35 min and cooled down to room temperature. The white precipitate was filtered off and washed with 50 mL water. The white powder (16.2 g) was received after drying *in vacuo* at $60~^\circ$C for 3 days.

#### Oxidation reaction

After swelling of the previously synthesised polymer (4.002 g, 17.78 mmol, 1.000 eq.) in 20 mL water for 20 min, 45 mL ethanol were added. The mixture was cooled to $0~^\circ$C and stirred for further 20 min. ${\hbox {H}_2\hbox {O}_2}$ (1.5 mL, 30%, 19.18 mmol, 1.079 eq.) was added to the mixture over a period of 1 h. Subsequently, EDTA (0.455 g, 0.16 mmol, 0.009 eq.) and ${\hbox {Na}_2\hbox {WO}_4\cdot2\hbox {H}_2\hbox {O}}$ (0.132 g, 0.40 mmol, 0.023 eq.) were added. First, two more equivalents of ${\hbox {H}_2\hbox {O}_2}$ (3.0 mL, 30%, 38.37 mmol, 2.158 eq.) were given to the mixture over the course of 2 h and stirred for additional 1 h, then, three more equivalents of ${\hbox {H}_2\hbox {O}_2}$ (4.5 mL, 30 $\%$, 57.55 mmol, 3.597 eq.) were added over the course of 1 h. Afterwards the mixture was warmed to room temperature and another three equivalents of ${\hbox {H}_2\hbox {O}_2}$ (4.5 mL, 30 $\%$, 57.55 mmol, 3.597 eq.) were added and stirred for 72 h. For removing excess ${\hbox {H}_2\hbox {O}_2}$ the blend was heated to $45~^\circ$C for 2 h. The precipitation was filtered off and dried *in vacuo* at $60~^\circ$C for 72 h. The reddish PTMA (3.3 g) was received.

### Electrode fabrication

PTMA-based electrodes were made by combining 300 mg PTMA (60 wt%), 175 mg SuperC65 (35 wt%) and 25 mg CMC (5 wt%) in water (2.5 mL) with a dispermat. The slurry was coated onto an aluminium current collector foil, which was previously cleaned with isopropyl alcohol, via doctor blading method (200 $\upmu {\hbox {m}}$ gap width). The layer was dried at room temperature for at least two days. After punching, the electrodes (diameter $d=12$ mm) were dried *in vacuo* at $65~^\circ$C for 2 days. The electrodes exhibited an averaged mass loading of 1.2 mg cm$^{-2}$.

### Electrochemical investigations

All electrochemical experiments were conducted using a CR2032 coin cell setup, which were assembled under inert atmosphere. PTMA-based electrodes (diameter of 12 mm, thickness of around 120 $\upmu {\hbox {m}}$) were used as cathode, Li metal (*Honjo*, diameter of 14 mm, thickness of 50 $\upmu {\hbox {m}}$) as anode and 50 $\upmu {\hbox {L}}$ LP57 (1 M ${\hbox {LiPF}_6}$ in EC:EMC 3:7) as electrolyte. One layer of *Celgard* 2500 separator was placed between the cathode and the anode side. Before the first cycling all cells were relaxed during a 12 h open-circuit voltage (OCV) step.

Cycling life performance tests were conducted on a *Biologic* VMP potentiostat. The cells were cycled between 3.0 V and 4.0 V with a constant current cycling mode. After three formation cycles at 0.2C (0.02 mA cm$^{-2}$) the cycling was performed at 1C (0.1 mA cm$^{-2}$) in a climate chamber at $20~^\circ$C. Rate capability tests were accomplished on a *Maccor* battery cell analysis system. During these experiments 1C equalled 0.1 mA cm$^{-2}$. Cyclic voltammetry investigations were carried out using a *Biologic* VMP potentiostat. The measurements were performed at a scan rate of 50 $\upmu {\hbox {V}}$ s$^{-1}$ and in a potential range of 2.5 V–4.5 V.

### EPR spectroscopy

#### Sample preparation

In an argon filled glove box, TEMPO methacrylate samples were dissolved in acetonitrile to generate a concentration series ranging from 1 mM to 300 mM. To prepare polymer solutions, 1 mg of PTMA polymer sample was dissolved in 200 $\upmu {\hbox {L}}$ of NMP. For EPR spectroscopy with solution samples, 20 µL of the sample solution were transferred to 2 mm outer diameter (OD) EPR tubes.

For PTMA spin counting experiments, powder samples were dried in an oven under reduced pressure (100 mbar) at 60 $^{\circ }$C for a week and 7 mg of the dried powder was then transferred to 4 mm OD EPR tubes under argon atmosphere. The EPR tubes were sealed under argon before EPR measurements.

The PTMA–CB sample was prepared by mixing PTMA polymer sample IV, suspended in toluene, and CB into a slurry to achieve a PTMA polymer IV to CB weight ratio of 2:1, followed by drying for two weeks at 60 $^{\circ }$C in an oven under air. The dried mixture was crushed into a fine powder using a mortar and pestle. 2 mg of the solid sample was transferred to a 2 mm OD EPR tube. TEMPO methacrylate–CB samples were also prepared using the same protocol.

A PTMA–CB–electrolyte sample (10 mg) was prepared by dry mixing PTMA polymer sample IV (60 wt%), SuperC65 (35 wt%) and CMC (5 wt%). The mixture was crushed into a fine powder using a mortar and pestle, followed by drying in an argon atmosphere for 17 hours at 60 $^{\circ }$C. The dry mixture was transferred to an EPR tube (4 mm OD) and 100 $\upmu {\hbox {L}}$ of the electrolyte (1 M ${\hbox {LiPF}_6}$ in EC/DMC = 1/1 (v/v)) was added to the sample within the EPR tube. The EPR tube was left undisturbed until the electrolyte fully permeated and soaked the sample, followed by sealing the tube under argon before EPR measurements.

#### Continuous-wave (CW) EPR

X-Band CW EPR spectra were recorded at room temperature as first derivatives of absorption spectra, using a *Bruker* EMX spectrometer operating at 9.65 GHz. All spectra were acquired with non-saturating microwave power of 0.3162 mW, with a modulation amplitude of 0.05 mT, equal to approximately one tenth of the narrowest line to avoid artificial broadening, and a modulation frequency of 100 kHz. For experimental $g_\mathrm{iso}$ determination, a 3-bisdiphenylene-2-phenylallyl (BDPA, *Bruker* calibration sample) standard ($g = 2.00254$) was used for field calibration. Spin counting of powder samples was done using Bruker Xenon software, version 1.3.

#### Pulsed EPR

All pulsed EPR experiments were implemented on an X-band *Bruker* ELEXSYS E580 with a *Bruker* EN 4118-X-MD4 pulse ENDOR resonator. The temperature was maintained with a helium cryostat (*Oxford Instruments* CF935). For experiments at cryogenic temperatures, the samples were flash frozen using liquid nitrogen and then inserted into the cryostat. Field swept echo detected EPR spectra were acquired using a standard two-pulse Hahn echo sequence with $\pi /2$ pulse length of 16 ns, $\pi$ pulse length of 32 ns and an inter-pulse delay $\tau$ of 200 ns. For $T_{1}$ measurements, an inversion recovery sequence ($\pi$ – *T* – $\pi /2$ – $\tau$ – $\pi$ – $\tau$ – echo) with 4-step phase cycle was used. A $\pi /2$ pulse length of 14 ns and $\pi$ pulse lengths of 28 ns were used. $\tau$ was kept at 200 ns. The delay *T* after inversion was set to an initial value of 400 ns and incremented linearly in steps of 2  $\upmu {\hbox {s}}$ to obtain a data set with 1024 recovery times. Laplace inversion of $T_{1}$ relaxation data was done using an exponential kernel without non-negativity constraint, with pre-processing of data and parametrization as described elsewhere.^35,44^ The inversion was performed using home-written scripts that were run on Octave v. 6.4.

### MD simulation

Classical molecular dynamics (MD) simulations^45^ were used to simulate the PTMA electrode in the presence of LP57. To understand the dynamics for different charge states of PTMA generated during the battery charging, three different cases were studied : i) No TEMPO radical units in the polymer were oxidised to TEMPO cations, ii) 50% TEMPO radical units were transformed to TEMPO cations, iii) All the TEMPO radicals units were oxidised. DFT based ${\textbf{g}}$ tensor calculations of the polymer structures indicated that a minimum of 6 monomer units was required to obtain a good agreement between experimental $g_\mathrm{iso}$ and DFT calculated $g_\mathrm{iso}$ (see Table S3). Therefore, for all three cases, six monomers were used to represent each of the PTMA polymers and a total of 24 of such polymers were used to mimic the electrode. For imitating the density of PTMA in LP57 used for electrochemical investigations, 1064 EC, 2100 EMC and 300 ${\hbox {LiPF}_6}$ molecules were chosen in a $10 \times 10 \times 10$ $\hbox {nm}^{3}$ simulation cell, with periodic boundaries in all three Cartesian coordinates. For 50% TEMPO cations, three out of six monomers of each polymer chain were chosen and, to make a charge-neutral simulation cell, a total of 72 ${\hbox {PF}_{6}^-}$ anions were added extra. Similarly for 100% TEMPO cations, a total of 144 ${\hbox {PF}_{6}^-}$ anions were added to make a charge-neutral cell. The initial configurations were constructed using PACKMOL^46^, which avoids repulsive potentials by keeping a safe inter-atomic distance.

All the MD simulations were performed using GROMACS 2019^47^. Before the equilibration step, a relaxation step was done for 2 ns with a 0.5 fs time step. In this relaxation step, both temperature and pressure were controlled with a Berendsen thermostat and a Berendsen barostat^45^, respectively, both with time constants of 1.0 ps. The reference temperature was taken as 298.15 K and the reference pressure as 100 bar. The Coulombic interactions were handled using a particle–particle particle–mesh (PPPM) solver with a cutoff of 1.2 nm interactions. For the equilibration step, a simulation for 20 ns with 1 fs time steps was performed. In this case, temperature and pressure were controlled with a Nosé–Hoover thermostat and a Parrinello–Rahman barostat, respectively, both with time constants of 1.0 ps. The reference pressure for this equilibrium step was 1 bar. The rest of the parameters were the same as the relaxation step. Finally for the collection of data, a 100 ns simulation with the exact same parameters as used for the equilibration step was run.

The OPLS all-atom force field^48^ was used for all the MD simulations. The atomic site charges on both TEMPO radical and TEMPO cation were calculated from the electrostatic potential (ESP) fit of methyl-terminated neutral and positively charged PTMA repeat units, respectively. Gaussian16^49^ was used to calculate the ESP charges using MP2 theory^50^ with pVDZ basis set. With the same method, the ESP charges of EC, EMC and ${\hbox {PF}_6^-}$ were calculated as well.

TEMPO structures (redox pairs) were taken from the MD trajectory and used for the electron hopping rate calculation. To calculate the electron hopping rates between two redox centres for typical distances, Marcus theory^18,19^ of electron transfer was used. The Marcus rate of electron transfer is described as

1 $$\begin{aligned} k_{\textrm{hop}}=\frac{H_\mathrm{RP}^2}{\hbar }\left[ \frac{\pi }{k_{\textrm{B}} T \lambda }\right] ^{1 / 2} \exp \left[ -\frac{\left( \lambda +\Delta G^{\circ }_\mathrm{RP}\right) ^2}{4 \lambda k_{\textrm{B}} T}\right] \,. \end{aligned}$$

$H_\mathrm{RP}$ is the electronic coupling and $\Delta G^{\circ }_\mathrm{RP}$ the free energy difference between the reactant state and the product state, $\lambda$ is the reorganisation energy, $\hbar$ the reduced Planck constant, and $k_{\textrm{B}}$ the Boltzmann constant. $H_\mathrm{RP}$ was calculated using the CASSCF/GMH method^51^ with pVDZ basis set (using ORCA v. 5.0.3^52^), and $\lambda$ was calculated using DFT with UB3LYP functional and 6-31++G(d,p) basis set (using Gaussian16)^19,21^. $\Delta G^{\circ }_\mathrm{RP}$ was considered to be zero due to the fact that all monomers are chemically identical.

The above-mentioned simulation parameters, force field and ESP fitting methods were also used to get the MD structures for the ${\textbf{g}}$ tensor calculations using DFT described in the next section.

### DFT-based EPR parameter calculations

The structures obtained from MD simulation trajectories were used for ${\textbf{g}}$ tensor calculation without further geometry optimisation. 10 PTMA chains out of 24 were randomly selected for each time frame. EPR parameter calculations using DFT were conducted using ORCA v. 5.0.2^52^. Geometry optimisation of the reference polymer structure with 6 monomer units was done at UKS/B3LYP level with a triple zeta basis set (def2-TZVP)^53^ without constraints on any atoms. Using a geometry optimised structure of TEMPO methacrylate (UKS/B3LYP/def2-TZVP), EPR calculations were done at UKS/B3LYP level with 6-31G(d),^54^ N07D^55^, EPR-II^56^ and EPR-III^57^ basis sets. B3LYP in combination with EPR-II gave $g_\mathrm{iso}$ values in good agreement with the experimental $g_\mathrm{iso}$ at the lowest computational cost. For calculation of the ${\textbf{g}}$-tensor of the geometry optimised reference polymer structure, UKS/B3LYP in combination with EPR-II basis set was used. A conductor-like polarisable continuum model (CPCM) was used for the calculation of NMP. The calculated $g_\mathrm{iso}$ was compared to the experimental $g_\mathrm{iso}$ of a linear PTMA polymer sample in NMP. Since ${\textbf{g}}$ is a gauge dependent property^58^ and since exchange was found experimentally for the polymer, different origins for ${\textbf{g}}$ were tested, yet for this system no significant gauge dependence was found. The origin of the ${\textbf{g}}$-tensor was set to the centre of spin density. EPR parameters of polymer structures from the MD trajectory were calculated at UKS/B3LYP level using EPR-II basis set with Resolution of Identity (RI) approximation and automatic generation of auxiliary basis sets.^59^

## Results and discussion

### Material synthesis

Four different cross-linked PTMA samples containing different amounts of PTMA-linked and free nitroxides were synthesised following and modifying an established procedure by Münch *et al.*^43^ (Fig. 1 and Table 1). In a first step, TMPMA monomer and EG-DMA cross-linker were reacted via emulsion polymerisation using sodium dodecyl sulfate as a surfactant agent. The resulting cross-linked polymer was oxidised with ${\hbox {H}_2\hbox {O}_2}$ to obtain the final PTMA polymer. ${\hbox {Na}_2\hbox {WO}_4\cdot 2\hbox {H}_2\hbox {O}}$ served as catalyst in this reaction. In total, roughly ten equivalents of ${\hbox {H}_2\hbox {O}_2}$ as oxidative agent were added to the intermediate polymer for the synthesis of PTMA samples labelled I, II and IV in order to receive a high amount of nitroxide radicals attached to the backbone. For PTMA sample III, only three equivalents of ${\hbox {H}_2\hbox {O}_2}$ were added to produce a lower fraction of active sites.

### Continuous wave (CW) EPR

**Figure 2:**
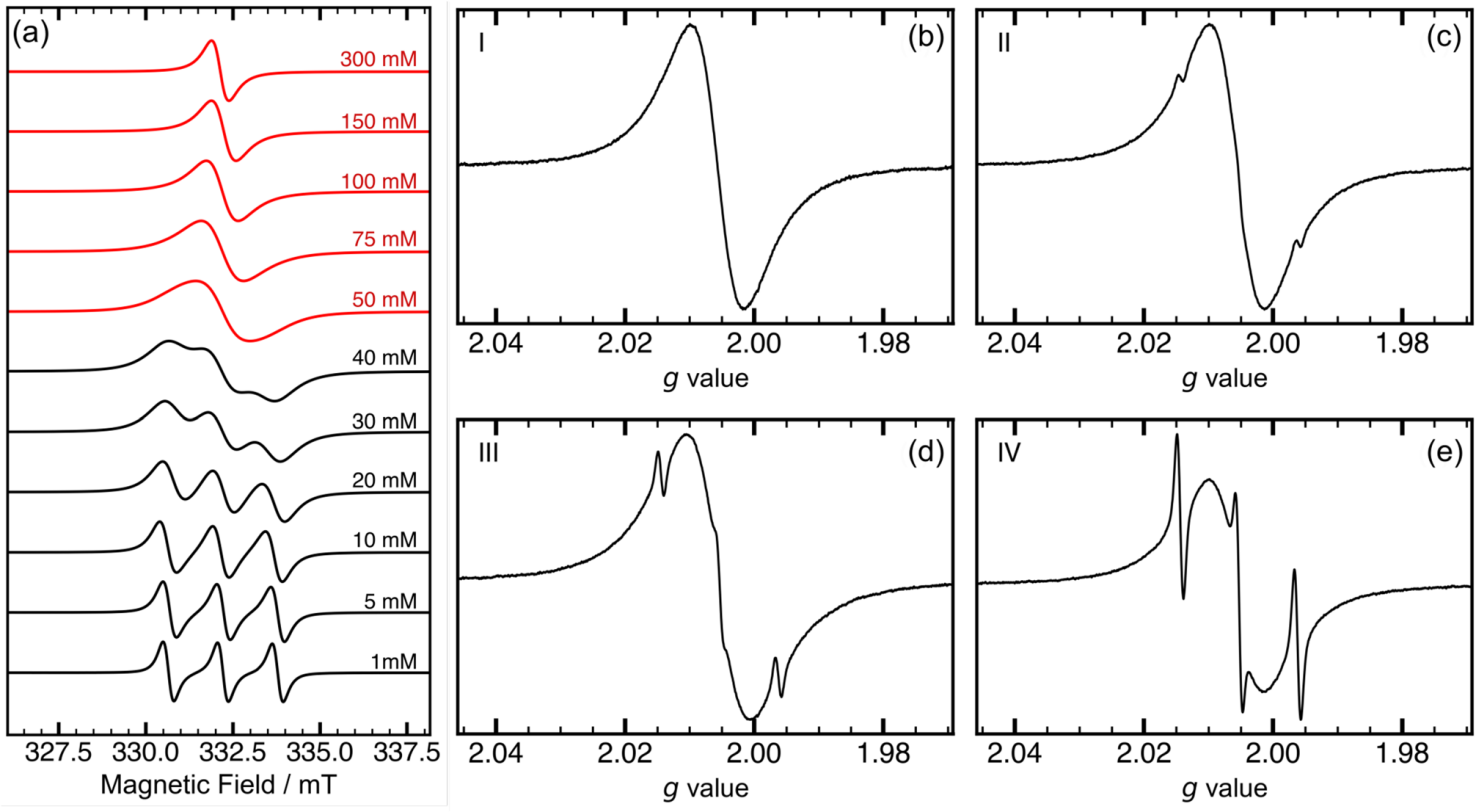
X-Band continuous wave EPR spectroscopy of PTMA at 295 K. (**a**) EPR spectra for varying concentrations of TEMPO methacrylate in acetonitrile. The concentration series can be divided into two regimes: The slow exchange (exchange broadening) regime (black) corresponding to the condition $k_\text {hop} \ll A_\mathrm{iso}$, and the fast exchange (exchange narrowing) regime (red), which corresponds to the condition $k_\text {hop}\gg A_\mathrm{iso}$. (**b**)–(**e**) EPR spectra of PTMA polymer samples in NMP with different amounts of isolated radicals. (**b**) Sample I, which showed no isolated radicals. (**c**) Sample II with 0.3% isolated radicals. (**d**) Sample III with 1.0% isolated radicals. (**e**) Sample IV with 2.6% isolated radicals. The contribution of isolated radicals to the total radical concentration was obtained by fitting the spectra using EasySpin^64^ (see ESI Tables S1 and S2).

Electron exchange plays a crucial role for the utility of PTMA polymers, where long-range charge transport and electrochemical performance depend on closely spaced redox units on the polymer chain. Since unpaired electron spin detected by EPR is located on the exchanging electrons, spin exchange becomes a direct measure for electron hopping processes. To demonstrate the effect of spin exchange and radical concentration, EPR spectra of TEMPO methacrylate (PTMA monomer) at different concentrations are shown in Fig. 2a. In solution, Heisenberg spin exchange occurs mainly through collisions between radicals that result in a considerable overlap of electron density.^60,61^ The exchange rate $k_\text {hop}$ is inversely proportional to the time $\tau _{d}$ between radical collisions and proportional to the probability $p_\mathrm{ex}$ of exchange taking place during a collision.^62,63^

At low concentration, the EPR spectrum consists of three lines arising from the hyperfine interaction of the unpaired electron with the $^{14}$N nuclear spin, split by isotropic hyperfine coupling constant $A_\mathrm{iso}$ (see Fig. 2a, black spectra). At high concentrations, the collision frequency $\tau _{d}^{-1}$ and, therefore, spin exchange increase. This manifests in the EPR spectrum initially as a line broadening of the three individual hyperfine lines, followed by a shift to the centre of the spectrum. As $k_\text {hop}$ exceeds $A_\mathrm{iso}$ at even higher concentrations, the strong exchange limit is reached and the lines coalesce to produce an exchange narrowed EPR spectrum (see Fig. 2a, red spectra). In this limit, an increase of the exchange interaction causes further line narrowing. Therefore, the linewidth of the EPR spectrum serves as an indicator of spin exchange and can be utilised to compare spin systems where spin exchange is prominent.

Fig. 2b–e shows the EPR spectra of PTMA polymers I–IV in NMP. The main contribution to each of the spectra arises from closely spaced nitroxide radicals, indicated by the broad EPR line. NMP causes swelling of the polymer and a solvation of the radicals tethered to the polymer. Thereby, spatial proximity is maintained due to the cross-linked polymer backbone, while spin exchange still predominantly occurs as in liquids. The linewidth of the broad component was extracted using lineshape analysis (see electronic supplementary information (ESI), Table S2). PTMA samples I, II and IV show similar peak-to-peak linewidths in the range of 1.3–1.4 mT for the broad component, indicating similar spin interaction strength. PTMA polymer sample III in NMP showed a broader linewidth of 1.6 mT, indicating a reduced spin exchange interaction. This may be attributed to the lower oxidation time during synthesis, resulting in less dense radical packing. The trend was similar for the corresponding powder samples, where the linewidth decreases to 1.0 mT for I, II and IV, and to 1.2 mT for III due to smaller radical–radical distances (see ESI Fig. S1). The dry powder polymer samples resemble more closely the case of rigid lattices, where dense packing causes a static overlap of electron density. The absence of swelling leads to a reduced mobility and a decrease in the configuration space sampled by individual radical centres. The polymer preferably adopts a compact conformation with increased nitroxide–nitroxide contact, as indicated by the increased exchange interaction. In this limit, increasing exchange interactions cause further line narrowing in comparison to polymers in NMP and, depending on the ratio between dipole–dipole interaction and exchange coupling, quenching of dipole–dipole broadening may occur.^65^ At the same time, line shapes become more Lorentzian, which was observed experimentally. If, on the other hand, powder conformations would be preferred with maximum distance between radicals, then the dry sample would likely experience additional broadening due to dipole–dipole interaction and reduced exchange interaction despite the absence of swelling.

If dipolar interactions are assumed to be quenched, the observed linewidth is proportional to $A_\mathrm{iso}^2/k_\text {hop}$^66^. The value of $k_\text {hop}$, calculated from the linewidths of powder spectra (see ESI Fig. S1), was found to be in the range of 38–45 MHz for the polymer samples, which is indicative of spin exchange in an intermediate regime when compared to $A_\mathrm{iso}$ of TEMPO methacrylate in NMP (see ESI Table S1). However, relaxation may additionally broaden the EPR line, hence the obtained $k_\text {hop}$ only represents a lower limit.

The observation that radicals I, II and IV show an equal line broadening despite a different number of spins indicates that a similar nitroxide clustering occurs in all three samples, with distances between clusters varying. For sample III, a reduced exchange interaction was observed. For the samples investigated here, this implies that the local conformation of radicals and electron hopping between them is more strongly affected by the synthesis protocol than by the overall density of radicals in the sample.

A distinguishing feature between the different polymer samples comprises the actual amounts of isolated radicals. These isolated radicals, which do not participate in spin exchange, exhibit a characteristic three-line EPR spectrum of nitroxides. Rotational correlation times, which can be estimated from the intensity ratio of the three lines,^67^ were found to be comparable to that of a dilute solution of the TEMPO methacrylate (1 mM) in NMP, suggesting that the isolated radicals are highly mobile, *i.e.* solvent accessible and not restricted by the polymer backbone. This could be the case if the isolated radicals result from incomplete polymerisation. The contribution from radicals undergoing exchange and isolated radicals are clearly distinguishable in the CW EPR spectra, indicating that the interaction among them is weak. As previously reported for linear PTMA^68^ and supported by the increased exchange narrowing of powder samples compared to polymers in NMP, the cross-linked polymer possibly adopts a compact conformation in solution, which remains inaccessible to the isolated radicals. This inference is significant, as an increase in isolated radicals, inaccessible to the primary spin exchange network, could be detrimental to long-range charge transport and, consequently, to battery performance.

### Electrochemical characterisation

**Figure 3:**
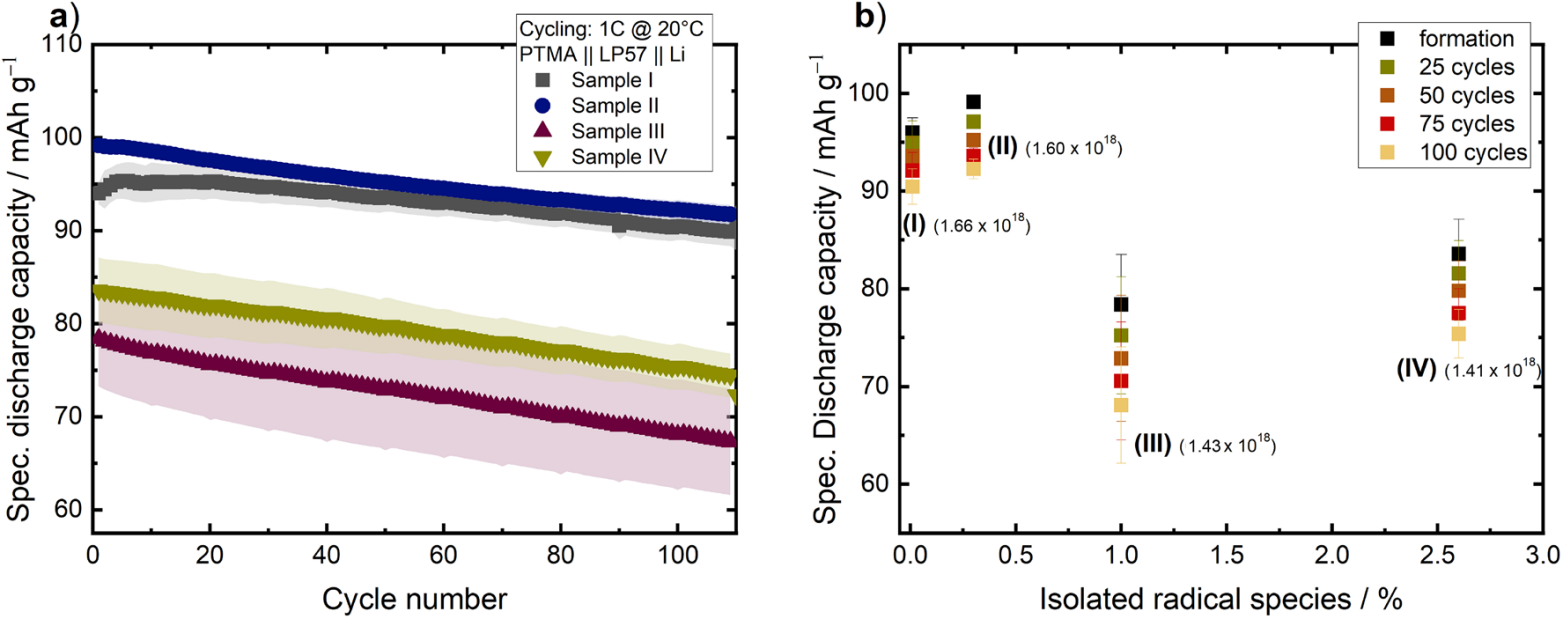
(**a**) Long-term cycling performance of $\mathrm {PTMA \,\vert \, LP57 \,\vert \, Li}$ coin cells at 1C up to 110 cycles. The data shown are average values of five cells each. (**b**) Specific discharge capacity of the four investigated PTMA samples with different amounts of isolated species after the formation (black), the 25th(green), 50th (orange), 75th (red) and 100th (yellow) cycle. Values in parentheses correspond to the total number of spins per mg for each sample obtained using spin counting CW EPR experiments.

To determine the electrochemical performance of the polymer samples and examine the impact of isolated radicals, several tests in a coin cell setup (CR2032) were conducted. The cells consisted of PTMA-based cathode, LP57 as electrolyte, one layer of Celgard 2500 as separator, and Li metal as anode. Long-term cycling is shown in Fig. 3a. For every cell, first a 12 h OCV step and three formation cycles at 0.2C (0.02 mA cm$^{-2}$ constant current charge and discharge) were performed before the actual cycling experiment at 1C (0.1 mA cm$^{-2}$ constant current charge and discharge) started. Sample I and II showed the highest initial specific discharge capacities of 96.0 mAh g$^{-1}$ and 99.1 mAh g$^{-1}$, respectively, and the highest State-of-Health (SoH) after 100 cycles. The values are in good agreement with previously reported PTMA investigations.^43^ In contrast, polymer samples III and IV showed lower initial discharge capacities of 78.4 mAh g$^{-1}$ and 83.6 mAh g$^{-1}$, respectively, and exhibited a more pronounced fading behaviour. For III and IV the SoH deteriorated at about twice the rate than for I and II.

For analysing the reversibility of the underlying redox process, cyclic voltammetry experiments at a low scan rate of $50~{\upmu \hbox {V}\,\hbox {s}}^{-1}$ were done. For all samples, one peak each for oxidation and reduction, which can be associated to the one-electron redox couple ${\hbox {NO}^{\mathbf{.}} / \hbox {NO}^{+}}$, could be observed (see ESI Fig. S6). Ratios of the anodic to the cathodic peaks of approximately 0.9 and peak-to-peak separations of 61–83 mV reflect a quasi-reversible redox process. Minor deviations from a fully reversible redox process (peak-to-peak separation of 57 mV^69^) indicate the presence of side reactions, *e.g.* between the cathode and the electrolyte. However, it should also be taken into account that overpotentials of the Li metal anode may shift the position of the redox peaks^70,71^. The rate capabilities of cells operated with the four samples at rates of 0.1C–50C revealed no significant difference in the rate performance (see ESI Fig. S7). All four samples exhibited nearly identical values for the capacity retention in the C-rate tests, even though the initial discharge capacities were different.

**Table 1:** Initial discharge capacities of $\mathrm {PTMA \,\vert \, LP57 \,\vert \, Li}$ coin cells prepared using the investigated PTMA samples I–IV, compared to corresponding number of spins per mg of sample and percentage of isolated radicals.

Sample	Number of spins per mg / x10$^{18}$	Isolated species / %	Initial $C_{disc.}$ / mAh g$^{-1}$	SoH after 100 cycles / %
I	1.66 ± 0.10	–	96.0	94
II	1.60 ± 0.16	0.3	99.1	93
III	1.43 ± 0.08	1.0	78.4	86
IV	1.41 ± 0.09	2.6	83.6	87

The initial discharge capacities of all polymer samples correlated with the number of spins determined by EPR (see Table 1). In case of polymer samples III and IV with similar total number of spins, a pronounced fading behaviour was observed. Capacity fading was minimal in I and II, attributed to a higher total number of spins. Correlation with the amount of isolated radicals is inconclusive (see Fig. 3b). Sample II with a low yet measurable amount of isolated radicals shows comparable cyclability as sample I with no isolated radicals detected. While samples III and IV contain higher amounts of isolated radicals than I and II, the pronounced fading behaviour cannot solely or conclusively be attributed to isolated radicals. This aspect is clear when the amount of isolated radicals in III and IV is compared, which differ by a factor three (see Table 1), yet the corresponding cells show similar SoH after 100 cycles (see Fig. 3).

For organic radical polymers, the origin of capacity fading during battery cycling is often linked to gradual dissolution of the active materials into the electrolyte. Free monomer radicals may readily dissolve into the electrolyte. To test for such a dissolution, the polymer active materials were suspended in toluene, in which cross-linked polymers exhibit minimal dissolution. After centrifuging, the supernatant was extracted for EPR analysis (see ESI Fig. S2). Sample I does not show a signal component originating from isolated monomer radicals. Instead, only a weak broad component with identical linewidth as the EPR line of the polymer is observed, indicating minor dissolution of the active material.

Spectral features of the supernatant spectra for polymer samples II, III and IV indicated that the dissolved active material consist primarily of isolated monomer radicals. Intensities are consistent with a quantitative washing out of radical monomers. Rapid dissolution of isolated radicals upon contact with electrolyte would lead to a drop of the initial capacity, but may not directly impact the long-term degradation of SoH. Also, electron hopping pathways that may be mediated by isolated radicals do not degrade gradually in case of fast dissolution. It is noted that in case of a real battery, binder may affect the dissolution of radicals by the electrolyte as well. However, free radicals in the binder would be electrochemically inactive, and binder should not impede the contact of active material and electrolyte, therefore such an effect is expected to be minor.

### Pulsed EPR

**Figure 4:**
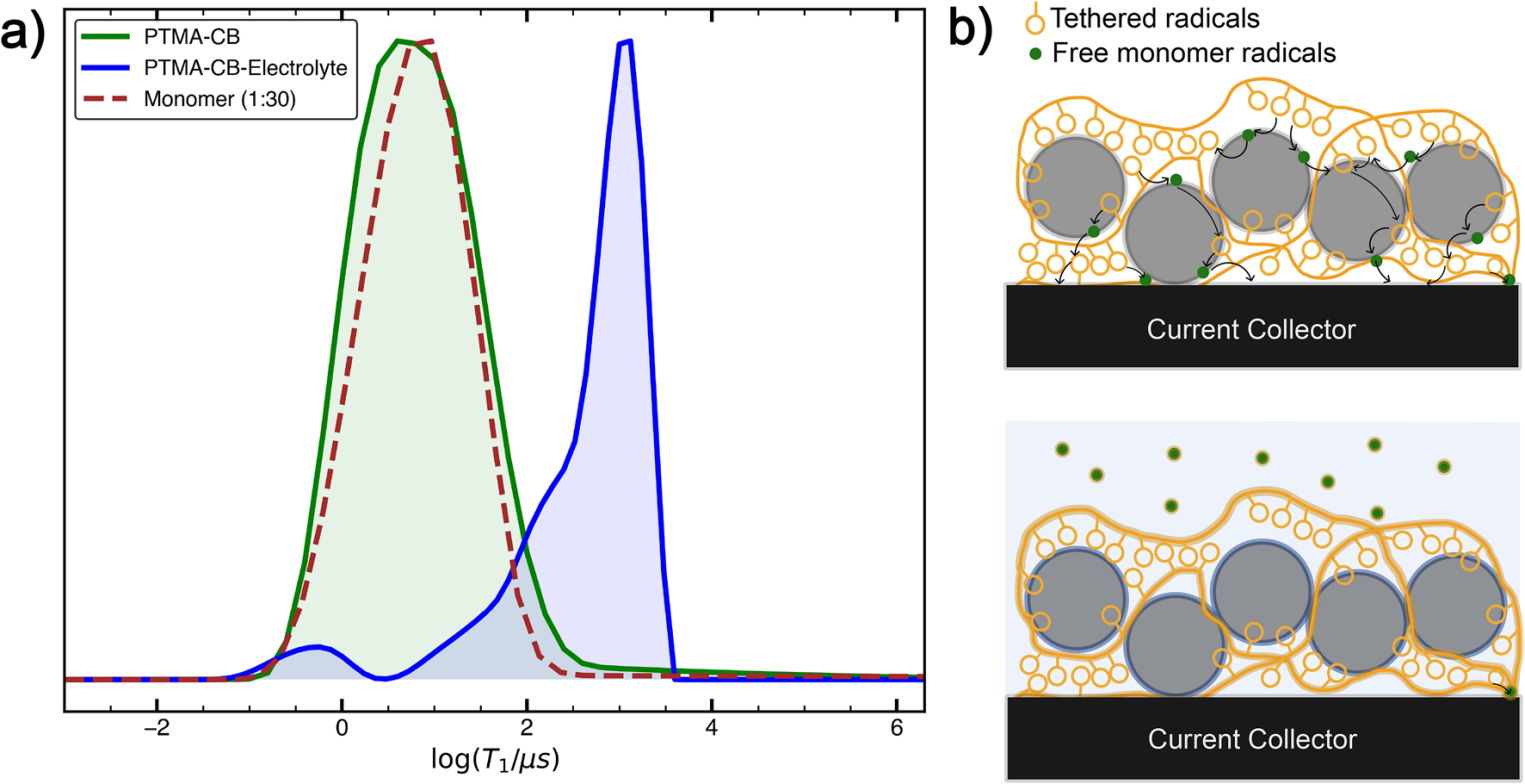
(**a**) EPR $T_1$ relaxation time distributions for PTMA–CB (green) and PTMA–CB–electrolyte (blue) samples, compared to a TEMPO methacrylate–CB (weight ratio of 1:30) reference sample (brown dashed). Inversion recovery experiments were conducted at 30 K and $T_{1}$ data were inverted using an exponential kernel to obtain the distributions. (**b**) Schematic representation of the distribution of monomer radicals (green dots) in the sample. Fast $T_1$ relaxation of the isolated radicals in PTMA–CB imply a good, direct contact with CB (top), with possible participation in electron hopping pathways (black arrows). A uni-modal distribution exhibited by the PTMA–CB, similar to the 1:30 monomer–CB sample, indicates that the isolated radicals are isotropically distributed on the CB surface. In case of PTMA–CB–electrolyte (bottom), the isolated radicals dissolve in the electrolyte, losing contact with CB and, consequently, exhibiting a slower $T_1$ relaxation.

Capacity fading linked to free monomer radicals is indicative for their participation in the electrochemical process or, equivalently, electron hopping. From CW EPR experiments, the free monomer radicals were found to be isolated from radicals tethered to the polymer. Therefore, the electron hopping between the isolated radicals and a neighbouring electron hopping site is likely aided by the carbon black conductive additive. To gain insight into the immediate environment of the isolated radicals and study their contact with CB, solid state samples analogous to the composition of the cathode were investigated by pulsed EPR in the absence of the electrolyte (see Fig. 4b (top) for a pictorial representation).

Polymer IV was chosen as the active material since it exhibited the largest fraction of isolated radicals. Pulsed EPR experiments are selective to the isolated radicals only, as the radicals undergoing exchange contribute negligibly to the spin echo for the selected echo time. The field-swept echo (FSE) detected EPR spectrum of PTMA–carbon black (PTMA–CB) sample was similar to the frozen solution spectrum of the TEMPO methacrylate (see ESI Fig. S4). In systems with a large concentration of dipole–dipole coupled spins, such as the polymers investigated in this work, instantaneous diffusion is expected^72^, which results in the decrease in intensity of the central $m_{I} = 0$ transition relative to the $m_{I} = 1$ and $m_{I} = -1$ transitions, where $m_I$ represents the magnetic quantum number of the ${^{14}\hbox {N}}$ nuclear spin. However, the FSE EPR spectrum did not indicate such interactions, hence the isolated radicals are spatially distant from nitroxides undergoing exchange.

To investigate the contact between CB and isolated radicals, spin–lattice relaxation time constants $T_{1}$ were measured. Figure 4a shows the $T_{1}$ distribution of a PTMA–CB sample compared to a TEMPO methacrylate–CB sample (1:30 TEMPO methacrylate to CB weight ratio). The $T_{1}$ distribution was obtained using inverse Laplace transform (ILT). The two samples showed almost identical relaxation characteristics, with a dominant relaxation mode at $T_{1} \approx 4~{\upmu \hbox {s}}$ and no slowly relaxing contributions. We recently reported such a pronounced relaxation enhancement for nitroxide radicals in direct contact with carbon black^35^. It suggests a good and direct contact of all the isolated radicals with CB, *i.e.* the isolated radicals show a high affinity for the CB surface. A good contact with CB raises the possibility of these isolated radicals to participate in electron hopping processes and, therefore, contribute towards the observed electrochemical capacity.

To explore the effect of electrolyte on the contact between isolated radicals and CB, the $T_{1}$ distribution of a PTMA–CB–electrolyte sample was determined. The addition of electrolyte led to a $T_{1}$ increase for isolated radicals by two orders of magnitude (see Fig. 4a). The relaxation distributions were also compared to the relaxation distribution of a PTMA polymer sample without CB (see ESI, Fig. S5), where a similar relaxation distribution to PTMA–CB–electrolyte was found. The minor component with $T_1 < 10~{\upmu \hbox {s}}$ observed in PTMA–CB–electrolyte, which was not present in the PTMA sample without CB, corresponds to a small fraction of radicals which remain in contact with CB. The obtained values are characteristic for isolated radicals in frozen solution, suggesting a loss of contact with CB in the presence of electrolyte. Nitroxide radicals that become more distant from the CB matrix experience reduced interactions with CB conduction electrons, leading to such a $T_{1}$ increase. In turn, a loss in contact between isolated radicals and CB leads to disconnected electron hopping pathways. Thereby, isolated radicals may no longer contribute to the redox reaction, resulting in capacity loss. Such a capacity loss could be direct, *i.e.* prevention of redox reactions on the radical monomers themselves, or indirect by disrupting monomer mediated contact between PTMA and CB. Another indirect mechanism arises from an increased contact area for the electrolyte between the PTMA–CB interface if monomers are removed from the surface. It would imply that the electrolyte could also disrupt the contact between PTMA and CB, but at a much slower rate than between monomers and CB. Since the capacity loss observed electrochemically is larger than the fraction of free monomers, a direct mechanism appears less plausible.

### Theoretical calculation of EPR parameters

**Figure 5:**
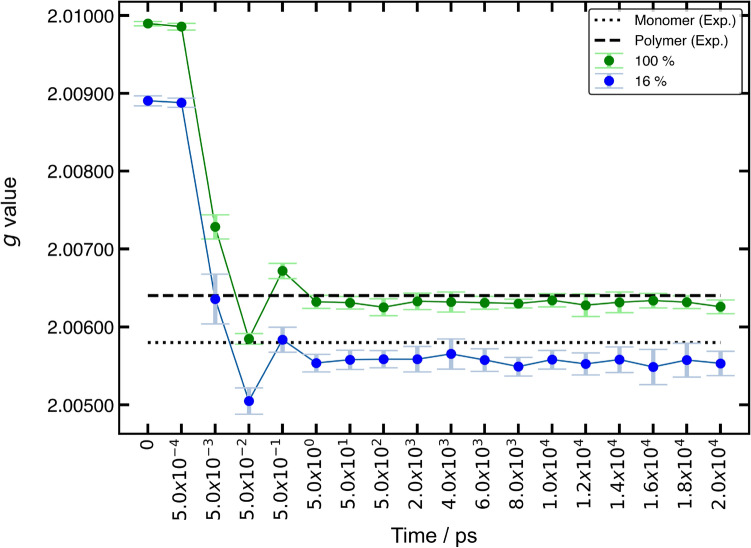
Validation of the MD methodology by DFT-based EPR parameter calculation. Convergence of calculated $g_\mathrm{iso}$ (filled circles) is compared to experimental values (black lines) as the simulation system equilibrates for 100% (green circles, dashed line) and 16% (blue circles, dotted line) radical density. $g_\mathrm{iso}$ values were calculated at B3LYP level of theory with EPR-II basis set using PTMA structures extracted from the MD trajectory. Each data point represents the average of isotropic *g* values of ten PTMA chains extracted from the same time frame of the MD simulation. Error bars denote the standard deviation.

Initially, MD simulation methodology used in this work was validated by comparing calculated with experimental *g* values. As a starting point, EPR ${\textbf{g}}$-tensors were computed using DFT methods. As a reference system for the validation, a linear PTMA oligomer with six monomer units was geometry optimised at B3LYP level and ${\textbf{g}}$ was computed for the optimised structure. The linear PTMA polymer sample in NMP was chosen as the experimental reference, with the experimental $g_\mathrm{iso}^\mathrm {(exp)} = 2.00641$. This value is also in agreement with previously reported *g* value of PTMA in ${\hbox {CH}_2\hbox {Cl}_2}$^73^. The calculated isotropic *g* value, $g_\mathrm{iso}^\mathrm {(calc)} = 2.00643$, was found to be in excellent agreement with $g_\mathrm{iso}^\mathrm {(exp)}$.

For MD protocol validation, EPR ${\textbf{g}}$ tensors were computed by DFT for PTMA polymer structures extracted from individual MD time frames without additional geometry optimisation. Fig. 5 shows the evolution of $g_\mathrm{iso}^\mathrm {(calc)}$ for PTMA polymer structures with 100% radical density (all monomer units are radicals) and for 16% radical density (one out of six monomers is a radical). Each data point represents the average $g_\mathrm{iso}^\mathrm {(calc)}$ for ten PTMA chains extracted from the same time frame of the MD simulation. The starting structure at $t = 0$ ps represents the unrelaxed geometry, which is corrected only with respect to inter-atomic distances and repulsive potentials. Consequently, a large deviation from the measured $g_\mathrm{iso}^\mathrm {(exp)}$ is observed. Nonetheless, the standard deviation for the starting structure is small, indicating that $g_\mathrm{iso}^\mathrm {(calc)}(t=0)$ is not a randomly scattered quantity. Instead it is sensitive to structural deviations and, hence, provides usable contrast regarding the consistency of a simulated PTMA conformation. During the relaxation run, which lasts until 2000 ps, $g_\mathrm{iso}^\mathrm {(calc)}$ approaches $g_\mathrm{iso}^\mathrm {(exp)}$ after $t \approx 5$ ps. In the equilibration run, ranging from 2000 ps to 20000 ps, $g_\mathrm{iso}^\mathrm {(calc)}$ converges, indicating an equilibrated structure.

The converged $g_\mathrm{iso}^\mathrm {(calc)}$ from the equilibration run was found to be 2.0063 for the 100% radical density case, which was within errors compared to $g_\mathrm{iso}^\mathrm {(exp)}$. For 16% radical density, TEMPO methacrylate in NMP was chosen as the experimental reference, with $g_\mathrm{iso}^\mathrm {(exp)} = 2.0058$. The average $g_\mathrm{iso}^\mathrm {(calc)}$ from the equilibration run of the 16% radical density case was found to be 2.0055. Such a deviation can be rationalised, as in the simulated structure the radical species is restricted to the polymer backbone whereas in the experimental reference, the nitroxide radicals are highly mobile in solution. Additionally, the MD structures were simulated in an electrolyte environment, which is considerably different from the solvent used in the EPR experiment. Calculations using a single TEMPO methacrylate molecule with an implicit solvation model for NMP gave a $g_\mathrm{iso}^\mathrm {(calc)} = 2.0057$ which was found to be in better agreement with the experimental reference in this case.

The remarkable agreement of $g_\mathrm{iso}$ values among the geometry optimised polymer structure, equilibrated MD structure, and experiment establishes the validity of the MD methodology. Furthermore, $g_\mathrm{iso}^\mathrm {(calc)}$ was found to be sensitive to the extent of equilibration of the polymer structure, making it a suitable parameter for similar validation studies.

### MD simulation

MD simulations of multiple PTMA oligomers immersed in LP57 were performed to characterise the interaction between PTMA chains and the constituents of the liquid carbonate electrolyte as well as the resulting ion dynamics. As the interactions generally depend on the charge of the polymers^74^, three different charge states were considered: i) 0% ${\hbox {TEMPO}^{+}}$, ii) 50% ${\hbox {TEMPO}^{+}}$ and iii) 100% ${\hbox {TEMPO}^{+}}$ units.

**Table 2:** Diffusivity of ions for different charge states of PTMA from the MD simulations.

Charge States of PTMA / % of $\hbox {TEMPO}^{+}$ in PTMA	$D_{\hbox {Li}^{+}}/ 10^{-7} ~{\hbox {cm}^{2}\,s^{-1}}$	$D_{\hbox {PF}_6^{-}}/ 10^{-7}~{\hbox {cm}^{2}\,s^{-1}}$	$D_{\hbox {PTMA}}$ $/ 10^{-8}~{\hbox {cm}^{2}\,s^{-1}}$
0	2.5	3.4	5.6
50	2.5	2.7	3.5
100	2.2	1.9	2.2

In Table 2, diffusivity of ${\hbox {Li}^+}$, ${\hbox {PF}_{6}^-}$ and PTMA have been compared for these three charge states. Diffusivities were calculated from the mean squared displacement (MSD) plots (see ESI Figs. S8, S9, S10). A significant decrease of the diffusivity of both PTMA and ${\hbox {PF}_{6}^-}$ by about 40–60% was observed when increasing the charge density on the PTMA chains, in contrast to only a marginal decrease of the ${\hbox {Li}^{+}}$ diffusivity. This can be attributed to the increase in coordination of ${\hbox {PF}_{6}^-}$ anions by increasing the amount of ${\hbox {TEMPO}^{+}}$ compared to the neutral ${\hbox {TEMPO}^{\mathbf{.}}}$, rendering both anions and polymer chains less mobile. Note that in the limit of long or even cross-linked chains the center-of-mass motion of PTMA becomes negligible. Therefore, in an experimental setup, the motion of the entire chain is likely irrelevant, even when applying an electric field.

The coordination behaviour can also be understood from the radial distribution function (RDF) of ${\hbox {PF}_{6}^-}$ with respect to the nitrogen atoms of TEMPO moieties (ESI Fig. S11). It is observed that the coordination number of ${\hbox {PF}_{6}^-}$ per nitrogen atom, calculated by integrating over the RDF peaks, increases from 1 to 3 while going from 0% to 100% ${\hbox {TEMPO}^{+}}$ moiety. Unlike ${\hbox {PF}_{6}^-}$, it was observed that ${\hbox {Li}^{+}}$ prefers to coordinate with neutral ${\hbox {TEMPO}^{\mathbf{.}}}$ (ESI Fig. S12).

**Figure 6:**
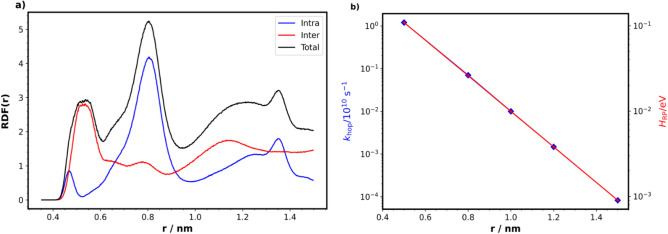
(**a**) Total RDF between nitroxide N nuclei of PTMA (black), split into contributions from intramolecular (blue) and intermolecular (red) radicals. Intra- and inter-molecular contributions of neighbouring N radicals are mainly originating from 0.68–0.94 nm and 0.45–0.6 nm peaks, respectively. By integrating the RDF for the respective peaks, average coordination numbers of neighbours per radical for intra- and inter-molecular contributions were found to be 1.45 and 0.28, respectively. (**b**) Distance dependence of Marcus rate ($k_\text {hop}$, blue diamonds) and the Marcus coupling ($H_\text {RP}$, red dots) is analysed for ${\hbox {TEMPO}^{+}}$–${\hbox {TEMPO}^{\mathbf{.}}}$ pairs.

From the RDF plots (Fig. 6a) between nitrogen radical centres of the PTMA polymers, it can be seen that dominantly two types of neighbouring peaks are present: a) the peak in the 0.45–0.6 nm range mainly corresponds to intermolecular neighbours, *i.e.* neighbours from different polymer chains, and b) the peak in the 0.68–0.94 nm range primarily corresponds to intramolecular neighbours, *i.e.* neighbours from the same polymer chain. Such a short intermolecular distance supports the formation of a compact polymer conformation. By integrating over the RDF peaks, average coordination numbers of neighbours per radical for intra- and inter-molecular contributions were found to be 1.45 and 0.28, respectively. Hence, the number of intramolecular neighbours is nearly five times higher than the number of intermolecular neighbours. Only about one in four nitroxides has a nearby intermolecular neighbour. Note that the chains in our simulations are short, such that we find less than two intramolecular neighbours on average.

In Fig. 6b (also in ESI Table S4), Marcus coupling ($H_\text {RP}$) and Marcus rates ($k_\text {hop}$) for various distances between nitrogen atoms of TEMPO units have been compared. Coupling values were calculated by averaging over all the orientations for certain distances. From our DFT calculation, reorganization energy ($\lambda$) was found to be 1.06 $\textrm{eV}$. This result is in agreement with previous studies done on electron hopping in PTMA^19,21^. From the resulting rates, it is observed that going from 0.5 nm (intermolecular contribution) to 0.8 nm (intramolecular contribution), the charge transfer rate is decreased by almost a factor 17, whereas from MD it was found that the average coordination number of neighbouring nitrogen atoms were only increased by a factor of five. Hence, from the product of these two factors, it may be expected that the overall charge hopping process is nearly 3.4 times more probable between intermolecular neighbours than intramolecular ones. On the other hand, since only every fourth nitroxide group is intermolecularly connected, long-range charge diffusion still requires more intra- than inter-molecular hopping events.

## Conclusions

Compositional characteristics of cross-linked PTMA polymers, which affect electron hopping processes, and their impact on electrochemical performance of a PTMA-based ORB were explored. EPR spectroscopy revealed that a major fraction of the radicals in polymer samples contribute to a broad feature of the EPR spectrum, indicative of spatially close radical units. A second component with a typical three-line spectral feature of dilute nitroxide radicals shows that a small yet measurable amount of isolated nitroxide units, which were not tethered to the polymer backbone, was also present in three of the four investigated polymer samples. Contributions from isolated radical monomers and radicals undergoing exchange could be quantified by CW EPR combined with lineshape analysis. It was found electrochemically that a small TEMPO methacrylate fraction on the order of 0.3% of the total nitroxide content did not show a considerable impact on capacity or SoH degradation. A quantitative effect for higher amounts of monomer radicals up to 3% was not found either, but a potential qualitative effect could not be excluded. Cycling rate performances were found to be similar despite the differences in the amounts of free monomer radicals.

For a mixture of PTMA polymer with carbon black, pulsed EPR showed that the free radical species was in good contact with CB. Such a contact indicates that as long as the isolated radicals do not undergo dissolution in the electrolyte, they may be electrochemically active via a carbon black assisted electron hopping pathway, either directly as redox units or indirectly to mediate hopping between CB and nitroxide units in the polymer. However, CW EPR indicated and pulsed EPR confirmed that free monomer radicals undergo dissolution in the electrolyte and lose contact with CB. This may indirectly influence the contact between CB and PTMA polymer in an electrode. Dissolution of monomer radicals that cover a significant fraction of the CB surface upon electrolyte contact may degrade the contact of CB with PTMA polymer or lead to an accelerated degradation.

MD simulations of PTMA in the presence of an electrolyte were validated by comparing calculated and experimental isotropic *g* values. Polymer structures extracted from MD trajectories were used for DFT based *g* value calculations. The calculated isotropic *g* values agreed well with experimental results obtained from EPR measurements. From the MD simulations, it was observed that with an increasing fraction of charged TEMPO units, the ${\hbox {PF}_{6}^-}$ diffusivity and the mobility of PTMA polymers decreased, while the mobility of ${\hbox {Li}^{+}}$ was only weakly affected. The distance dependence of electron hopping between two redox centres inside the PTMA electrode was also quantified. The analysis of electron hopping rate dependence on the structures from the MD simulations illustrated that electron hopping is more feasible among intermolecular neighbours compared to intramolecular ones. Since spin relaxation appears to be a major contribution for the observed exchange-narrowed EPR lines, exchange rates cannot be directly obtained from EPR linewidths. Nonetheless, they represent a lower limit for exchange rates in an exchange-narrowed system, which are consistent with calculated hopping rates.

Based on pulsed EPR data, the isolated radicals exhibit a high affinity for the CB surface, and a similar affinity may exist for the nitroxide radicals tethered to the polymer backbone. The observation that the isolated radicals are quantitatively located on the CB surface, even while a much larger amount of PTMA polymer was present, may be attributed to kinetics, since the isolated radicals show a much higher local mobility, as indicated by CW EPR. On the other hand, MD simulations suggest, supported by CW EPR results, that the polymer preferably adopts a compact conformation to increase the nitroxide–nitroxide contacts. Therefore, a competition may evolve between CB–nitroxide interactions and nitroxide–nitroxide interactions that determines the polymer conformation. Such a competition may have a direct impact on the electrochemically observed capacity fading. However, explicitly quantifying such a competition is challenging and will require systematically varied samples to be investigated electrochemically and spectroscopically. In addition, modelling will have to incorporate a CB surface to quantitatively capture the equilibrium. This may be particularly relevant since MD simulations suggested a significant variation of PTMA mobility upon battery cycling. Thereby, the polymer may progressively evolve towards a thermodynamically more stable conformation, amplified by a repetitive mobility alteration during charge/discharge cycles. Cycling between a conformation dominated by Coulomb interactions between ${\hbox {TEMPO}^{+}}$ and ${\hbox {PF}_{6}^-}$ ions in the charged state and weaker van der Waals interactions in the discharged state may help overcome activation barriers, conceptually similar to a freeze–thaw cycle. Such a thermodynamically more favourable conformation may be different from the initial kinetically influenced structure and, notably, may not be the electrochemically most active one. A higher density of radical groups may be better suited to maintain electron hopping in an evolved structure, explaining the electrochemically observed correlation between radical concentration and cycling stability. Overall, this signifies the necessity for a better understanding of electron transport in ORBs to further optimise both polymer synthesis protocols and active material compositions.

## Supplementary Information

Supplementary Information.

## Data Availability

The data sets used and analysed during the current study are available from the corresponding author on reasonable request.
