# Supplementary material for: Multimodal investigation of electronic transport in PTMA and its impact on organic radical battery performance
Source: Sci Rep. 2023 Jul 6;13:10934. doi: 10.1038/s41598-023-37308-5 (PMC10326018; doi:10.1038/s41598-023-37308-5)
Supplement: Supplementary file 1 — Supplementary Information. [file 41598_2023_37308_MOESM1_ESM.pdf]

Electronic supplementary information

Multimodal investigation of electronic transport in PTMA and its impact on organic radical battery performance

Davis Thomas Daniel[1,2], Steffen Oevermann[3,+], Souvik Mitra[4,+], Katharina Rudolf[5], Andreas Heuer[3,4], Rüdiger-A. Eichel[1,6], Martin Winter[3,5], Diddo Diddens[3], Gunther Brunklaus[3] and Josef Granwehr[1,2,*]
[1] Forschungszentrum Jülich GmbH, Institute of Energy and Climate Research (IEK-9), 52425 Jülich, Germany
[2] RWTH Aachen University, Institute of Technical and Macromolecular Chemistry, 52056 Aachen, Germany
[3] Forschungszentrum Jülich GmbH, Helmholtz Institute Münster (IEK-12), 48149 Münster, Germany
[4] University of Münster, Institute of Physical Chemistry, 48149 Münster, Germany
[5] University of Münster, MEET Battery Research Center, 48149 Münster, Germany
[6] RWTH Aachen University, Institute of Physical Chemistry, 52056 Aachen, Germany
[*] j.granwehr@fz-juelich.de
[+] These authors contributed equally to this work

CW EPR spectroscopy

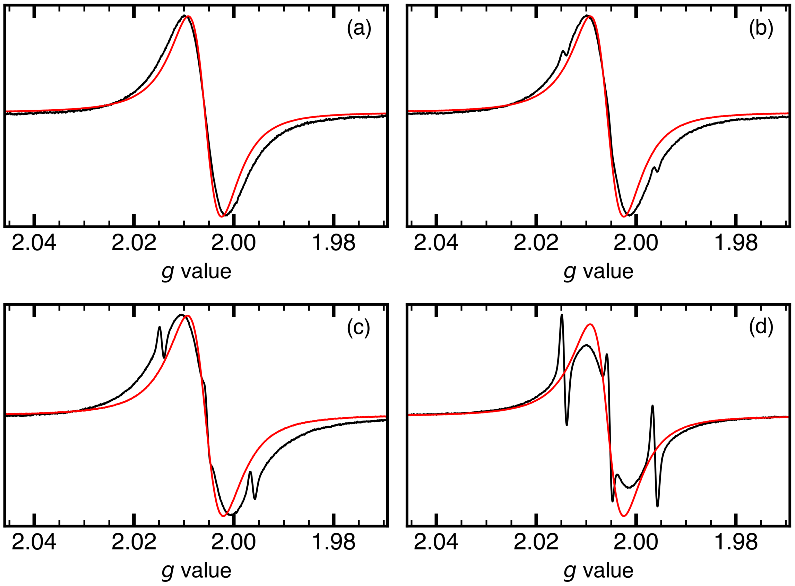

Figure S1. Comparison of X-Band continuous wave solution EPR spectra of PTMA polymer samples I (a), II (b), III (c), and IV (d) in NMP (black) and the corresponding powder EPR spectra (red). Solution state as well as powder spectra were recorded at 295 K.

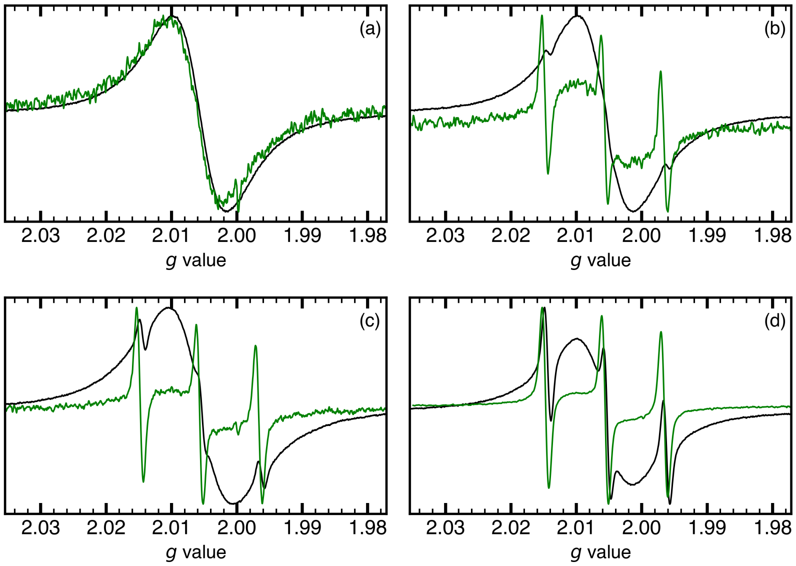

Figure S2. X-Band continuous wave EPR spectra of PTMA polymer samples in NMP at 295 K with different amounts of isolated radicals (black). The spectrum of isolated species (green) is extracted by suspending the polymers in Toluene, followed by centrifuging and collecting the supernatant for EPR spectroscopy. (a) PTMA sample I; (b) PTMA sample II; (c) PTMA sample III; and (d) PTMA sample IV.

Fitting of CW EPR spectra

The EasySpin software package was used to fit the spectra of PTMA polymer samples (I–IV) and extract the contribution of isolated radicals to the total spectra in each case (Figure S3). Two spin systems, corresponding to the isolated nitroxides (species **1**) and and nitroxide undergoing exchange (species **2**) were defined. For both **1** and **2**, initial values for **g** were obtained from DFT calculated **g** using geometry optimised structures of TEMPO methacrylate and PTMA polymer. In case of **1**, A_{iso} was read from the spectrum directly and used as an initial parameter. Lorentzian lineshapes were used for both **1** and **2** with isotropic convolutional broadening. The weights for **1** and **2** were initialised as 0.1 and 0.9 respectively. For fitting the CW EPR spectrum of polymer sample I, only species **2** was used. The fitted parameters are summarised in Table S1 and Table S2.

Polymer sample	g_{iso}	A_{iso} / MHz	Linewidth / mT	Weight
II	2.0059	43.6	0.17	0.003
III	2.0059	43.7	0.21	0.010
IV	2.0058	43.7	0.14	0.026

Table S1. Fitted parameters for species **1**

Polymer sample	g_{iso}	Linewidth / mT	Weight
I	2.0063	1.34	1.000
II	2.0064	1.43	0.997
III	2.0061	1.58	0.990
IV	2.0063	1.43	0.974

Table S2. Fitted parameters for species **2**

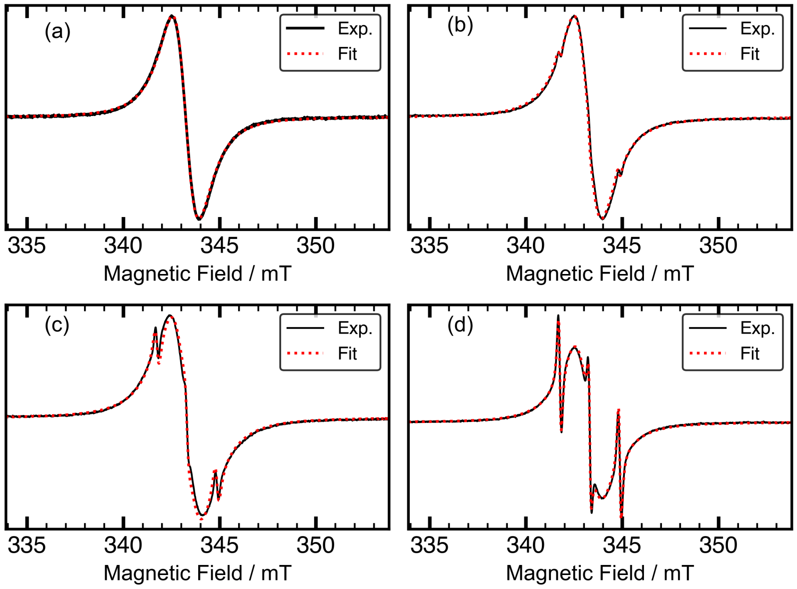

Figure S3. X-Band continuous wave EPR spectra of PTMA polymer samples (black, solid line) in NMP at 295K with different amounts of isolated radicals and corresponding fits (red, dotted line).

DFT calculations of g_{iso}

Number of monomers	$\langle g_{iso} \rangle$
2	2.00584 ± 0.00004
4	2.00609 ± 0.00004
5	2.00624 ± 0.00004
6	2.00638 ± 0.00004
7	2.00646 ± 0.00004

Table S3. Average g_{iso} values, calculated for PTMA polymer structures with a varying number of monomer units. $\langle g_{\text{iso}} \rangle$ was calculated from mean of g_{iso} values corresponding to structures extracted from MD frames at 0.5, 5, 50, 500 , 5000, 10000, 15000 and 20000 ps for each chain length. Convergence was attained for six monomer units, which represented a feasible compromise for computational cost of DFT calculations.

Marcus rate calculations

Distances between N atoms of TEMPO units/ nm	H_{RP}/eV	$k_{\text{hop}}/10^{10}\ \text{s}^{-1}$
0.5	0.109	1.2
0.8	0.026	0.07
1.0	0.009	0.01
1.2	0.004	$1.47\text{x}10^{-3}$
1.5	0.0009	$8.25\text{x}10^{-5}$

Table S4. Charge transfer rate between a TEMPO^{+} and a $\text{TEMPO}^{\bullet}$. From DFT, $\lambda = 1.06\text{eV}$.

Pulsed EPR

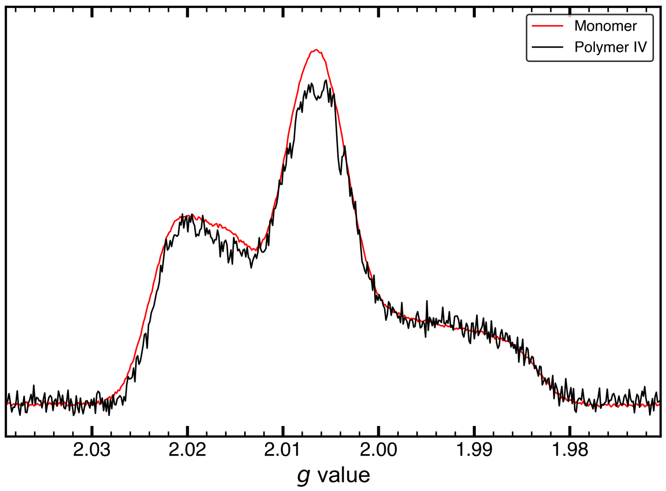

Figure S4. Field-swept echo (FSE) detected EPR spectrum of PTMA-CB sample compared to a FSE EPR spectrum of 1mM solution of PTMA monomer at 40 K.

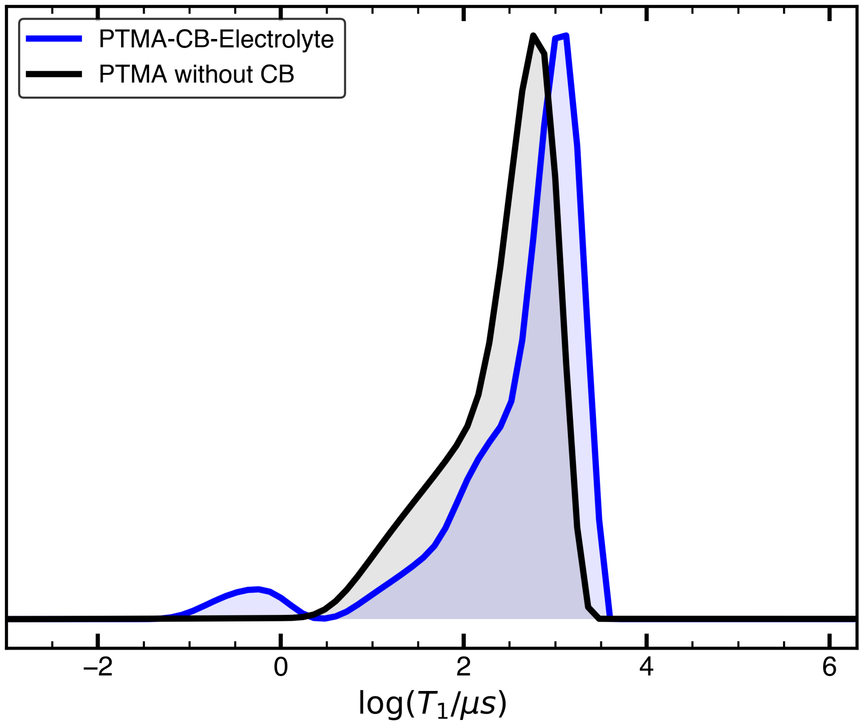

Figure S5. EPR T_1 relaxation time distributions for a PTMA–CB–electrolyte sample (blue) compared to a PTMA polymer sample without CB (black). The minor component with $T_1 < 10$ μs in PTMA–CB–electrolyte corresponds to a small fraction of radicals that remain in contact with CB.

Electrochemical characterisation

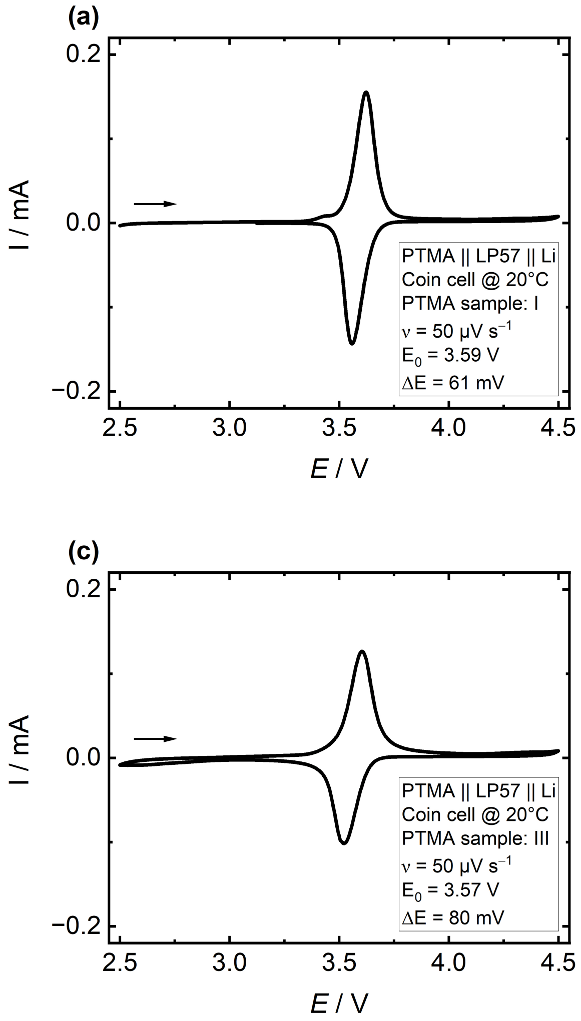

Figure S6. Cyclic voltammograms of PTMA|LP57|Li full cells in a coin cell setup at a scan rate of 50 µVs^{-1} and a potential range of 2.5-4.5 V. (a) PTMA sample I, (b) PTMA sample II, (c) PTMA sample III, and (d) PTMA sample IV.

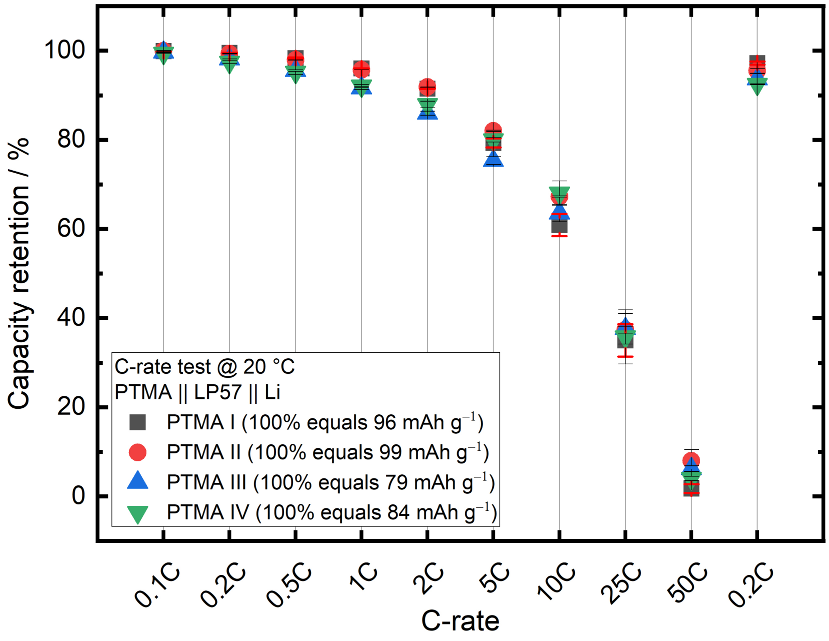

Figure S7. Rate capabilities of the four PTMA samples in coin cell setup PTMA|LP57|Li at 0.1C–50C. The shown data refer to three cells each, measured four times at each C-rate. Beforehand three initial formation cycles were performed for every cell at 0.2C.

Molecular Dynamics

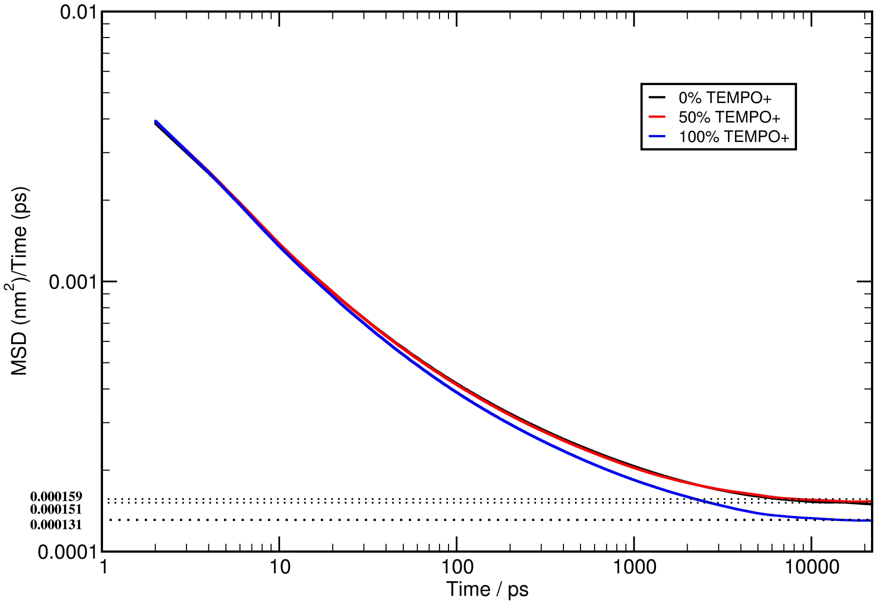

Figure S8. MSD/time *vs.* time plots for Li^+. Diffusion coefficients were calculated as $D = \frac{\text{MSD}}{6t}$.

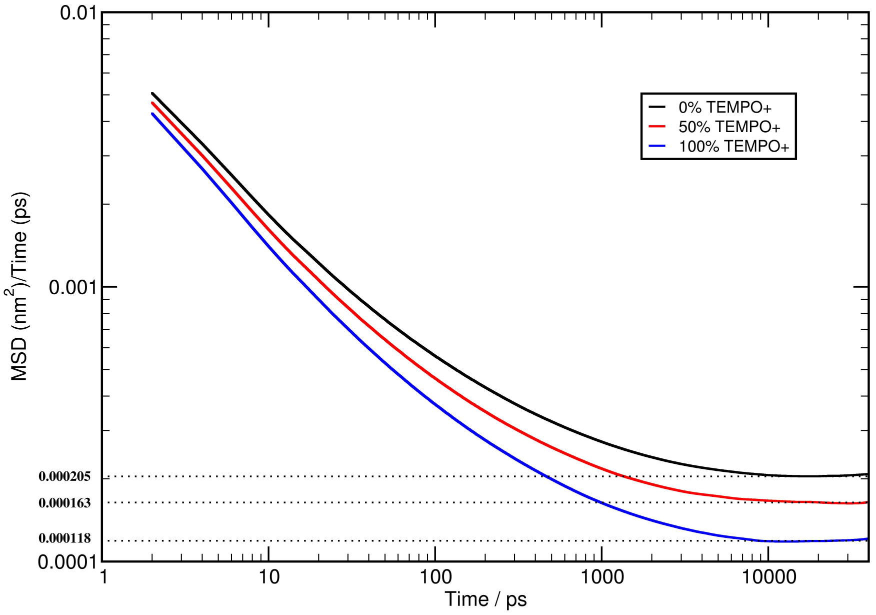

Figure S9. MSD/time *vs.* time plots for PF_6^-. Diffusion coefficients were calculated as $D = \frac{\text{MSD}}{6t}$.

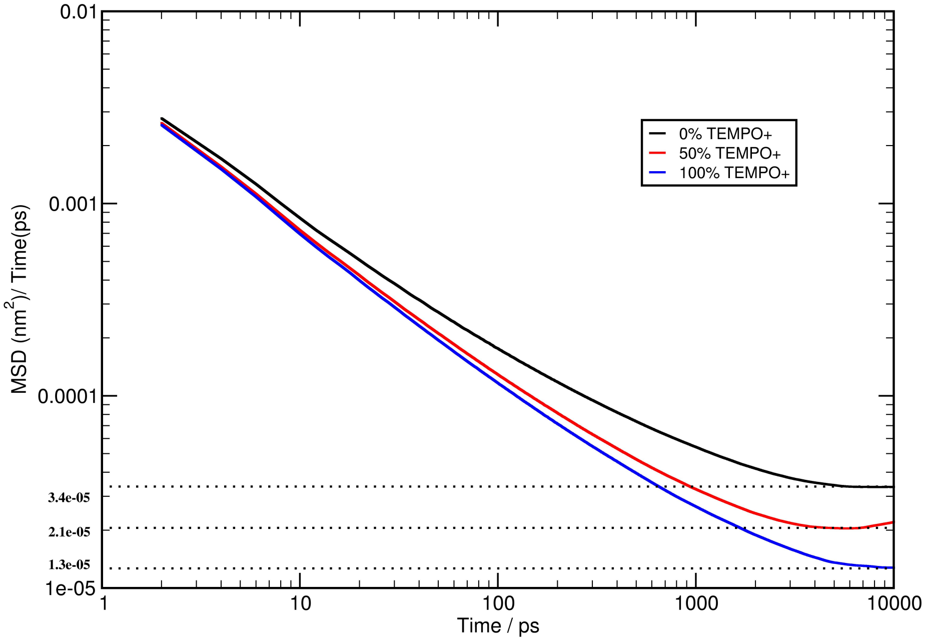

Figure S10. MSD/time *vs.* time plots for PTMA. Diffusion coefficients were calculated as $D = \frac{\text{MSD}}{6t}$.

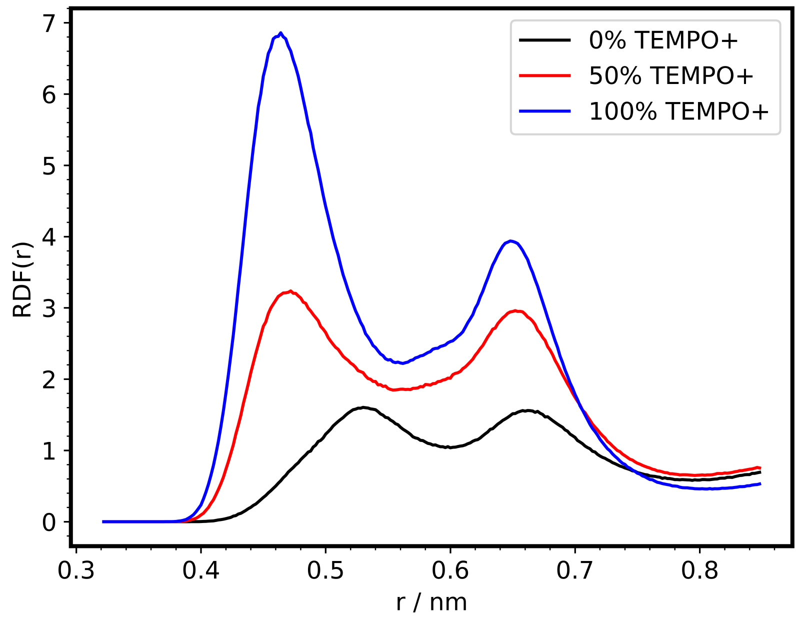

Figure S11. RDF of PF_6^- with respect to nitroxide N atoms of PTMA for 0 % $TEMPO^+$ (black), 50 % $TEMPO^+$ (red) and 100 % $TEMPO^+$ (blue). Integrating over the peaks, the average coordination number for PF_6^- per N atoms of PTMA were found to be 1, 2 and 3 for 0 % $TEMPO^+$, 50 % $TEMPO^+$ and 100 % $TEMPO^+$, respectively.

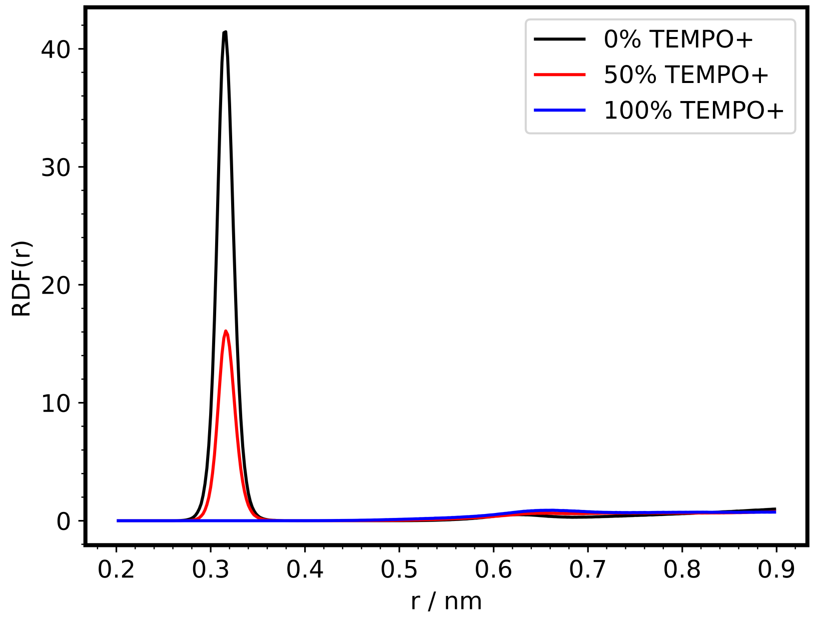

Figure S12. RDF of Li^+ with respect to nitroxide N atoms of PTMA for 0 % $TEMPO^+$ (black), 50 % $TEMPO^+$ (red) and 100 % $TEMPO^+$ (blue). Integrating over the peaks (0.28-0.36 nm), the average coordination number for Li^+ per N atoms of PTMA were found to be 0.6 and 0.25 for 0 % $TEMPO^+$ and 50 % $TEMPO^+$, respectively. No Li^+ coordination were found for 100 % $TEMPO^+$.
